# Artificial Intelligence in the Hands of Perfusionists: Revolutionizing Cardiopulmonary Bypass

**DOI:** 10.21470/1678-9741-2024-0005

**Published:** 2024-09-03

**Authors:** Glory Mini Mol Alexander

**Affiliations:** 1 Manipal Academy of Higher Education, Manipal College of Health Professions, Manipal, Karnataka, India. E-mail: gloryevangalin003@gmail.com

In the past decade, there have been remarkable technological advancements and innovations that have significantly influenced in the healthcare industry, now we are entering in a new era of medical practice, research, and patient care. Cardiac surgery has already reached significant milestones in the realm of robotic surgery, but the horizon of medical innovation steps into a new frontier: artificial intelligence (AI). As cardiac surgeons have increasingly embraced robotic techniques for intricate procedures, AI stands ready to revolutionize the field by offering unparalleled insights, decision support, and enhanced patient care. Just as robotic surgery once marked a transformative shift in cardiac surgical practices, the integration of AI holds the promise of further elevating the standards of care and outcomes in cardiac surgery^[1]^. In the ever-advancing landscape of healthcare, the intersection of AI and cardiac surgery has sparked new hope and innovation. Cardiac surgery, a field that demands the highest precision and safety standards, is being transformed by the integration of AI.

Perfusionists play a critical role in cardiopulmonary bypass (CPB), a procedure used during cardiac surgeries to temporarily take over the functions of the heart and lungs. Their responsibilities include maintaining the heart-lung machine, monitoring vital parameters, adjusting blood flow, and managing various parameters to ensure the patient’s safety during surgery. Traditionally, these tasks have been performed manually, relying on the perfusionist’s expertise and experience^[2]^.

AI will empower cardiac surgeons with enhanced decision support by analysing extensive patient data, including medical records, imaging, and real-time monitoring. This AI-driven analysis assists surgeons in making informed decisions during all phases of patient care, from initial diagnosis to postoperative management. It serves as a valuable tool in surgical planning, creating 3D models of a patient’s heart. These models offer an unprecedented level of precision in visualizing the heart’s anatomy, enabling surgeons to plan procedures with remarkable accuracy. Continuous real-time monitoring is a cornerstone of AI’s role in cardiac surgery. By monitoring a patient’s vital signs and medical parameters, AI can instantly identify deviations from the normal range and provide timely alerts to the surgical team. This vigilance helps prevent complications during surgery and provides critical data for quick decision-making, enhancing patient safety. As we know each patient is unique, and AI leverages this diversity by analysing individual characteristics, medical history, and current data. This personalized approach tailors treatment plans to meet the specific needs of each patient. Consequently, patients receive interventions that are not only more effective but also aligned with their unique healthcare requirements^[3]^.

The application of AI in perfusion, the field of providing mechanical circulatory support during cardiac surgery or other medical procedures, is an area of growing interest and innovation. AI is being utilized to enhance various aspects of perfusion technology and patient care. AI can facilitate real-time monitoring which is it can continuously monitor patient vital signs, blood flow parameters, and other relevant data during perfusion. It can analyze this data in real time and provide alerts or recommendations to perfusionists in case of deviations from the normal range. This assists perfusionists in making informed decisions and ensures early intervention if complications arise. AI algorithms can optimize the management of blood flow and pressure parameters during CPB. By adjusting these parameters in real time, AI can help maintain optimal conditions for the patient, reducing the risk of complications during surgery. AI can be integrated with electronic health record systems to provide seamless access to patient data. This integration allows for a more comprehensive view of a patient’s medical history, enabling better decision-making during perfusion^[4]^.

A case study reported that in the context of elderly, frail patients, the application of AI represents a robust approach with the potential to enhance the quality of care. Specifically, an AI system that continuously and autonomously electronically records health data to generate alerts offers a screening method characterized by its accessibility and ease of implementation. By enhancing the accuracy of delirium diagnosis and prompting essential interventions more consistently for this patient demographic, there is a strong likelihood of substantial improvements in the overall quality of care, and by extension, a measurable positive impact on patient outcomes^[5]^.

In conclusion, the integration of AI into the field of perfusion and, more specifically, CPB, marks a transformative shift in the landscape of healthcare. AI serves as an invaluable ally to perfusionists by providing enhanced decision support, optimizing surgical planning, and facilitating real-time monitoring during CPB procedures. It also brings automation to routine tasks, ushers in a new era of personalized patient care, and contributes to improved postoperative care, patient outcomes, training, and education, error reduction, data analysis, research, and cost-efficiency. As AI in perfusion continues to evolve, it is poised to redefine the standards of patient care and surgical outcomes. It is an exciting journey that promises to yield significant advancements in healthcare, and as we embrace this technological revolution, we must do so with the utmost commitment to patient safety, ethical practice, and the ongoing pursuit of excellence in cardiac surgery. The future of AI in the hands of perfusionists is one filled with promise and potential, and it is a journey that will continue to shape the landscape of healthcare for years to come.
